# Substance Use in Humanitarian Settings: A Case from Yemen

**DOI:** 10.1186/s13011-024-00606-w

**Published:** 2024-05-24

**Authors:** Ebtesam A. Saleh, Mayyada Wazaify, Kaveh Khoshnood

**Affiliations:** 1 Department of Psychiatry and Psychotherapy, Charité Universitätsmedizin, Campus Charité Mitte (CCM), Charitéplatz 1, 10117 Berlin, Germany; 2 Department of Pharmacology and Toxicology, Faculty of Pharmacy, Aden University, Aden, Yemen; 3 Department of Biopharmaceutics and Clinical Pharmacy, School of Pharmacy, The University of Jordan, Amman, Jordan; 4 Department of Epidemiology of Microbial Diseases, Yale School of Public Health, New Haven, CT USA

**Keywords:** Public health, Substance use, War, Yemen

Yemen has been suffering the worst humanitarian crisis in the world for over a decade [1]. In addition to the ongoing civil war, collapsed health system, starvation, and pandemics, Yemen has been hosting forcibly displaced populations; 4.5 million internally displaced populations and around 100,000 refugees and asylum seekers who came from Somalia, Ethiopia, Iraq, Syria, Eritrea and Palestine [1]. Evidence has shown that people affected by armed conflict, including forcibly displaced populations, are at high risk of substance use as a coping mechanism for emotional and psychological problems, and it should be a top priority to address the problem of substance use disorders (SUDs) in such humanitarian settings [2]. In addition to the psychological distress due to conflict, coping with idleness due to unemployment, inability to continue education, and poverty in a humanitarian context are risk factors of SUDs [3]. The major deterioration in all life aspects due to long-term war has impacted the mental health status of the population, yet this problem is still neglected in Yemen.

Moreover, the comorbidity of substance use and mental illness among the forcibly displaced population is becoming a growing concern that can have long-term medical, psychological, and legal ramifications [3].

Addressing the problem of SUDs in Yemen is still ignored. To date, there is no national data on the prevalence and extent of substance use among the distressed population in such a humanitarian setting [4]. However, several studies reported increased consumption of different types of substances (e.g., Khat, prescription, and non-prescription drugs), and due to the overall humanitarian situation in Yemen, addressing substance use is not a high priority for the government. Hence, there is no effective action to conduct further studies or establish a treatment strategy [5]. Illicit drugs such as methamphetamine and cocaine have also been recently reported in Yemen [6]. Nevertheless, there is no specialized treatment service or treatment strategy for substance use in Yemen. Only one center for rehabilitation of substance use and other psychological disorders was established in the capital, ‘Sanaa’ over a decade ago. However, it is not properly active due to the deterioration of the health system caused by the war. There is also an insufficient number of mental healthcare providers, and the number of specialized psychotherapists is 0.2% per 100,000 Yemeni, while the suggested minimum threshold is above 1 psychiatrists per 100,000 populations. Additionally, healthcare providers reported lack of information and training to identify and manage the problem of SUDs [4].

The problem of substance use in Yemen might be similar to other humanitarian settings as a result of coping mechanisms to trauma; however, the situation is further complicated in Yemen by the use of ‘Khat,’ a ‘natural amphetamine-like substance.’ Although it has serious adverse effects on the cardiovascular and nervous systems, Khat is socially and legally accepted in certain Horn African countries and Yemen. It is commonly consumed during social gatherings and perceived as a safe herbal energy booster for mental and physical activities [7]. Anecdotal reports suggest Khat is consumed for flu symptoms as well due to its pseudoephedrine-like effect and some users prefer to mix it with analgesics [5]. It is consumed even among children and pregnant women, resulting in major health consequences and a high mortality risk due to cardiomyopathy and heart failure associated with Khat consumption [7]. The problem worsens with mixing Khat with prescription drugs and other psychoactive substances. This has been reported to cause behavioral and mental health problems such as physical dependence, violence, crimes, and psychosis [5].

In addition to the aforementioned major health consequences, the inclines of crime levels might also be associated with substance use, considering that Yemen is the second most armed country in the world and weapons (e.g., machine guns, revolvers, fully automatic firearms) are legal among the population [8]. Accordingly, the problem of SUDs in this humanitarian setting, the ‘Yemeni context,’ is a more complex crisis with unpredictable consequences on the health and social levels among different age groups and genders of the community.

Healthcare providers need to enhance their expertise and capabilities in identifying and managing SUDs in similar humanitarian situations. Therefore, integrating training on substance use and SUDs in the curricula of healthcare faculties and colleges is of high priority. This can help primary healthcare professionals conduct further assessments and referrals for SUD treatment and help community pharmacists be the first line of defense and play a significant role in SUD prevention, education, and awareness [9]. In addition, a public health strategy for SUD management among war-affected populations in Yemen is highly needed, considering that SUD is a health condition affecting individuals, families, and communities. Providing mental health and psychosocial support, community engagement and peer-support, and integration of substance use services into primary healthcare. These public health approaches should involve a comprehensive and coordinated effort that prioritizes prevention, harm reduction, and treatment [10].

## Data Availability

No datasets were generated or analysed during the current study.

## Abbreviations

SUDs: Substance Use Disorders
